# Comparing survival outcomes between surgical and non-surgical treatments in patients with early-onset endometrial cancer and developing a nomogram to predict survival: a study based on Eastern and Western data sets

**DOI:** 10.1186/s12957-025-03825-y

**Published:** 2025-05-11

**Authors:** Yunfeng Zheng, Ran Hu, Fan Yang, Gaohua Liu, Tianyu Peng, Langting Xie, Jie Wu, Lamei Hou, Rui Yuan

**Affiliations:** 1 Department of Gynecology, The First Affiliated Hospital of Chongqing Medical University, Chongqing, 400016 China; 2 Department of Gynecology, Fengdu People’s Hospital, Chongqing, 408200 China; 3 Centre for Lipid Research & Chongqing Key Laboratory of Metabolism On Lipid and Glucose, Key Laboratory of Molecular Biology for Infectious Diseases (Ministry of Education), Department of Infectious Diseases, the Second Affiliated Hospital, Chongqing Medical University, Chongqing, 400016 China; 4 Institute of Clinical Medicine, Hengyang Medical School, The First Affiliated Hospital, University of South China, Hengyang, Hunan 421001 China; 5 Department of Gynecology and Obstetrics, Fujian Medical University Union Hospital, Fuzhou, Fujian 350001 China; 6 Department of Gynecology, People’s Hospital of Fengjie, Chongqing, 404600 China

**Keywords:** Endometrial cancer, early-onset, Nomogram, Overall survival, Risk stratification, SEER

## Abstract

**Background:**

Surgery is the preferred approach for treating endometrial cancer (EC). However, the prognosis of young women undergoing surgery has not been thoroughly evaluated. This study aims to establish a prognostic nomogram for predicting overall survival (OS) in postoperative patients with early-onset endometrial cancer (EOEC), facilitating risk stratification for high-risk patients.

**Methods:**

Patients diagnosed with EOEC during 2004–2015 were extracted from the Surveillance, Epidemiology, and End Results (SEER) database. The nomogram of OS was established according to the multivariate Cox regression analyses. The prediction accuracy and clinical net benefit of the model were assessed by the concordance index (C-index), receiver operating characteristic (ROC) curves, calibration plots, and decision curve analysis (DCA). Additionally, external validation was performed with 230 EOEC patients who underwent primary surgical treatment at the First Affiliated Hospital of Chongqing Medical University from 2013 to 2018.

**Results:**

The mean survival period in the surgical group of EOEC was 87.62 months (range: 86.92–88.32), compared to 64.00 months (range: 55.05–72.96) in the non-surgical group. Compared with the non-surgical group, patients who underwent surgery had better outcomes. A total of 4345 eligible postoperative patients with EOEC were identified and enrolled in this study. Multivariate Cox analysis showed that age, race, grade, T stage, tumor size, and lymphadenectomy were significantly associated with the prognosis of EOEC, which were further incorporated to construct a nomogram. C-index and DCA showed the predictive capability and the clinical applicability of the nomogram was superior over the TNM stage and SEER stage. Furthermore, the external validation using the FAHCQMU cohort consistently demonstrated good predictive accuracy.

**Conclusions:**

Generally, we developed a novel nomogram model by comprehensively integrating multiple risk factors, which accurately predicts the clinical prognosis of EOEC patients after surgery.

**Supplementary Information:**

The online version contains supplementary material available at 10.1186/s12957-025-03825-y.

## Introduction

Endometrial cancer (EC) is the most common gynaecological malignancy, and the sixth most common cancer in women, with an estimated 66,570 newly diagnosed cases and over 12,940 deaths in 2021, seriously threatening women’s health [1]. The overall incidence of EC is slowly increasing, while the number of young women with EC is doubled [2]. This may be due to the increased prevalence of EC screening and the influence of factors such as obesity [3], metabolic syndrome [4, 5], insulin resistance [6], and reproductive factors [7]. A woman’s lifetime risk of developing EC is about 3%, with a median age at diagnosis of 61 years old [8]. Patients with EC usually have classical clinical manifestations of postmenopausal bleeding, which facilitates early diagnosis. However, the pre-operative diagnosis of EC in premenopausal young women presents a clinical challenge due to the lack of specific biomarkers, reliance on histopathology, and non-specific symptoms.

There is no consensus on the explicit definition of early-onset endometrial cancer (EOEC). According to previous literature and clinical studies, EOEC denotes that EC patients are diagnosed at an age younger than 50 years old [9–11]. EOEC patients have a more favorable prognosis than elderly patients, with a higher frequent of well-differentiated tumors and better tumor stages [12]. However, recurrence can still occur even in early-stage patients with EOEC, which is the primary cause of cancer death.

In clinical practice, the FIGO and TNM staging systems have been widely implemented in the management of EC. Since the FIGO staging update in 2009, significant progress has been made in the understanding of the genetic diversity and drivers of the different pathogenic statuses of EC. The molecular classifications (*POLEmut*, MSI-H, CN-H, and CN-L) have been incorporated into the updated 2023 staging system [13, 14]. However, the high cost of molecular detection technology, elevated technical requirements, and the current unclear understanding of the molecular characteristics of EOEC pose challenges [15]. Additionally, the existing prognostic scoring system only considers tumor invasion, regional lymph node involvement, and distant metastasis as predictors, without incorporating demographic and clinical characteristics (e.g., obesity, hormone receptor status, genetic predisposition). This limitation makes the system inadequate and reduces its accuracy as a prognostic prediction tool [14, 16]. Therefore, it is necessary to develop nomogram models based on multiple risk factors to analyze the prognosis of cancer patients as a supplement and reference for EOEC risk stratification.

Currently, some nomograms have been constructed to predict the prognosis of EC patients; however, these models are not suitable for assessing the survival of EOEC due to their lack of specific risk factors for EOEC or because they were designed primarily for older EC patients [17, 18]. To fill this research gap, we conducted this study based on the SEER database to explore the prognostic variables and construct a specific nomogram for postoperative patients with EOEC. Then, the predictive performance and application value of the nomogram were validated and further compared with TNM stage and SEER stage. Effective prediction of prognosis in EOEC can inform evidence-based interventions for the individual and reduce the healthcare burden of this disease.

## Material and methods

### Data retrieved from SEER

**Fig. 1:**
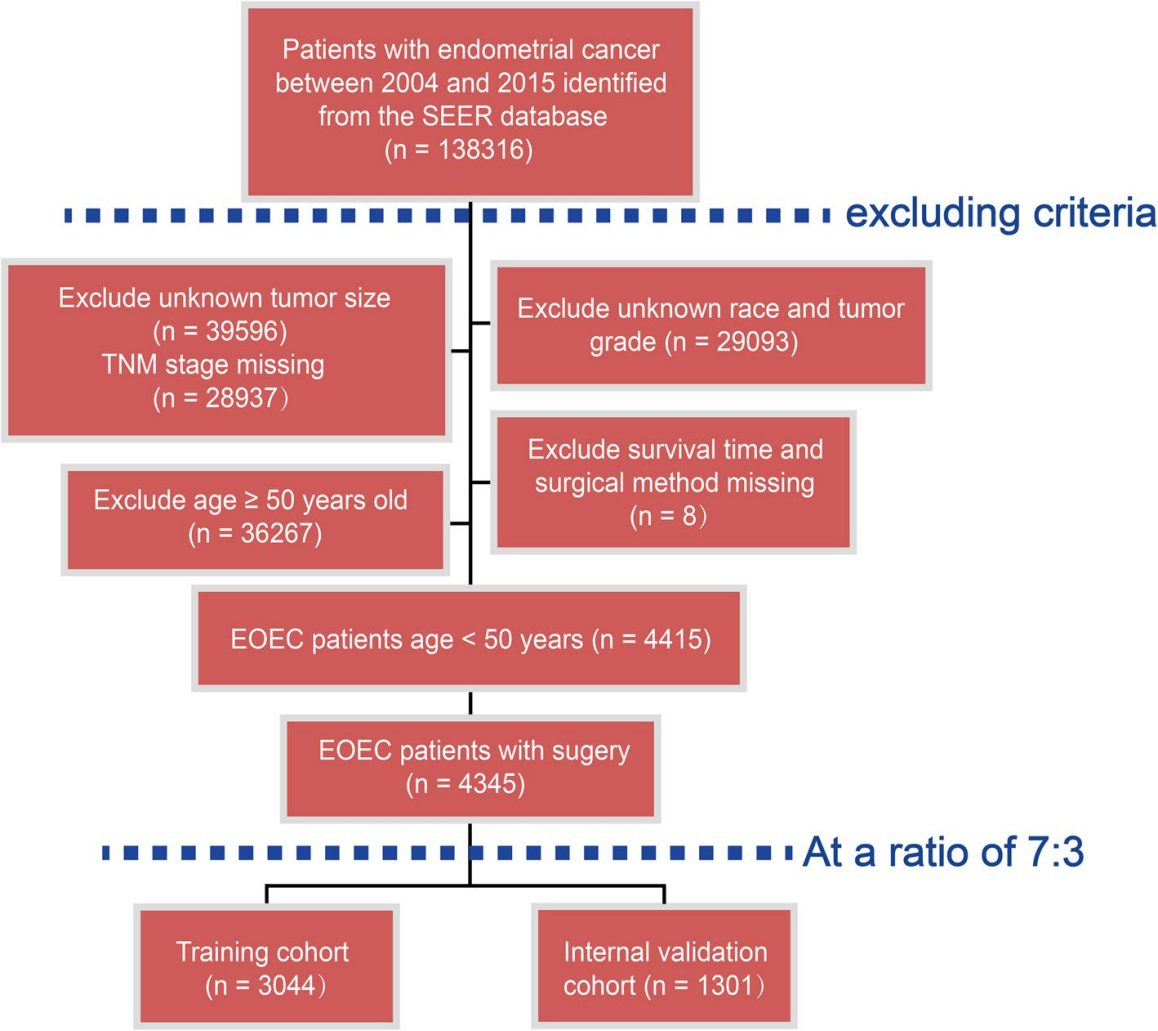
Flowchart of selection of EOEC

EC patients who were diagnosed from January 1, 2004 to December 31, 2015 were obtained from the SEER database using the SEER*Stat program (v8.4.0.1). Patients with EOEC were identified by the International Classification of Diseases for Oncology, third edition (ICD-O-3), and the cancer staging scheme (v0204) [19]. The exclusion criteria were (1) age ≥ 50 years; (2) multiple primary tumors; (3) incomplete demographic, clinical pathological data, including race, tumor size, grade, and TNM stage; (4) missing survival information or surgical records. The flow diagram of the screened EOEC patients was depicted in Fig. 1.

### External validation cohort

The clinical and pathological data of 230 EOEC patients who underwent primary surgical treatment between October 20, 2013 and May 20, 2018 at the First Affiliated Hospital of Chongqing Medical University were retrospectively analyzed. The inclusion criteria for patients were as follows: (1) age < 50 years; (2) patients with a definitive diagnosis of endometrial cancer based on the final pathological examination; (3) primary tumor; (4) complete medical records and postoperative follow-up information. The exclusion criteria were as follows: (1) age ≥ 50 years; (2) multiple primary tumors; (3) incomplete clinicopathological data, including tumor size, grade, and TNM stage; (4) incomplete medical records and postoperative follow-up information. The follow-up deadline for this study was May 20, 2021, and each patient was guaranteed a follow-up period of more than 3 years. Supplementary Fig. 1 illustrates the flow diagram of the screened EOEC patients.

### Variables

Variables encompassed age, race, grade, tumor size, TNM 7th stage, SEER stage, lymphadenectomy, radiotherapy, and chemotherapy. Tumor grade was defined as I (well differentiated), II (moderately differentiated), and III/IV (poorly differentiated or undifferentiated). OS was defined as the period from diagnosis to death or last follow-up. The X-tile program was utilized to evaluate the optimal thresholds for variables (tumor size and patient’s age) (Supplementary Fig. 2) [20].

### Statistical analysis

Patients with EOEC enrolled in this study were randomly assigned to a training cohort and a validation cohort using R software, adhering to a predefined ratio of 7:3. And, we use the ‘createDataPartition’ function for partitioning, which performs stratified sampling based on the target variable's distribution to ensure that the training and validation sets have a similar proportion of the target variable, achieving balanced distribution. After partitioning, we further checked the balance of the dataset. The training cohort was employed to develop the nomogram model, and then the accuracy of the prediction model was assessed using both internal and independent external verification cohorts. Detailed R code was provided in Supplementary Materials R [21]. Categorical data were expressed as frequencies with percentages, and further analyzed by Chi-square test or Fisher’s exact test. Kaplan–Meier analyses were utilized to generate and describe OS curves. Multivariate Cox regression was utilized to verify the predictors related to OS. Meanwhile, hazard ratio (HR) were calculated and displayed with 95% confidence intervals (95% CI). All statistical analyses were performed utilizing the SPSS software (version 25.0) and R (version 4.2.1; http://www.r-project.org/) software. *P*-value < 0.05 was considered statistically significant.

## Results

### Demographic and clinical characteristics

**Fig. 2:**
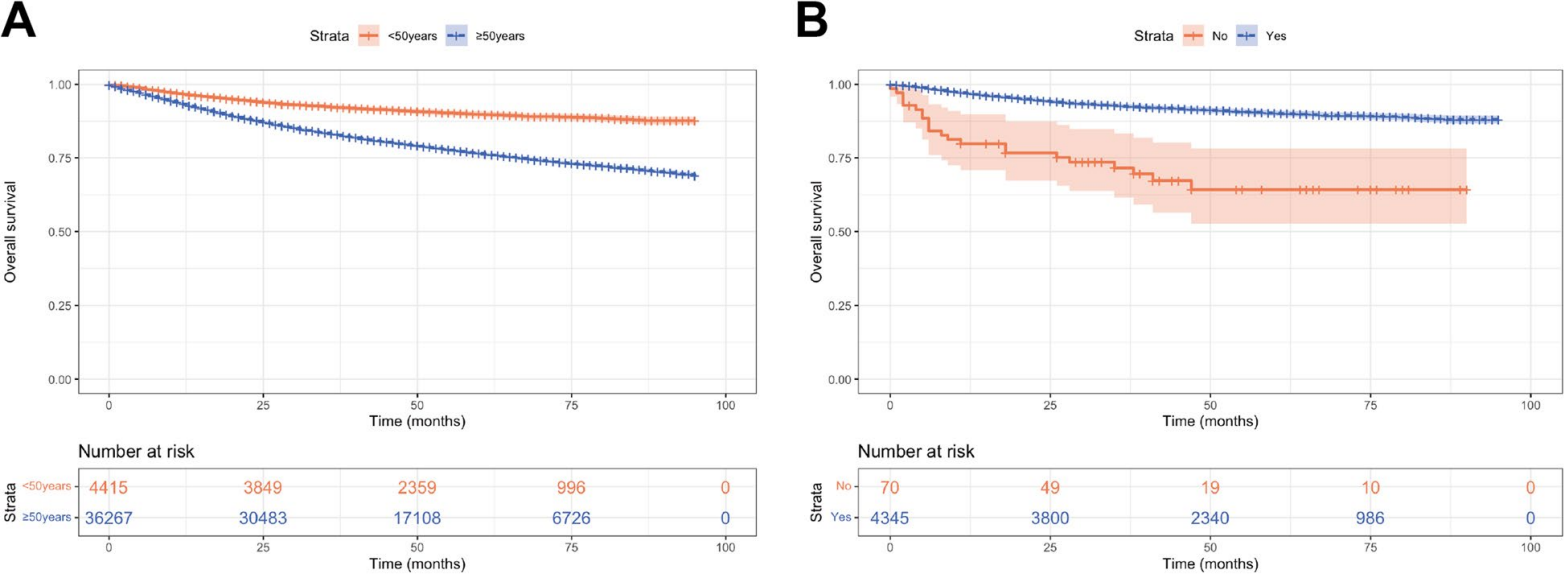
Kaplan–Meier plots of (**A**) OS in younger and elderly patients with EC; and (**B**) OS of EOEC patients who underwent surgical treatment and those who did not

Demographic information and clinical data of EC patients were selected from the SEER database during 2004–2015. A total of 4,415 younger and 36,267 elderly patients were screened and enrolled. Detailed information about clinical pathological features was demonstrated in Table 1. In the whole study cohort, elderly patients accounted for the majority of the cases (89.1%), whereas young patients accounted for only 10.9%. In terms of tumor grade and TNM staging, young patients had higher proportions of grade I (55.9%), T1 stage (81.5%), N0 stage (91.1%), and M0 stage (95.9%). Concerning treatment, more elderly patients underwent lymph node dissection (68.2%), as well as postoperative adjuvant therapy (40.7%). Moreover, survival analysis illustrated that younger patients had higher OS rates than those of elderly patients (*P* < 0.001, Fig. 2A); with regard to surgical treatment, patients who underwent surgery among the young patients had significantly better prognosis compared to those who did not receive surgical treatment (*P* < 0.001, Fig. 2B). Further analysis revealed that the mean survival period was 87.62 months (range, 86.92–88.32 months) in the surgical group, while the mean survival period was 64.00 months (range, 55.05–72.96 months) in the non-surgical group. The OS probabilities for the surgical group were 99.8%, 92.6%, and 90.2% at 1, 3, and 5 years after surgical treatment, respectively. For the non-surgical group, the OS probabilities were 79.8%, 71.6%, and 64.2% at 1, 3, 5 years, respectively (Supplementary Table 1).

**Table 1:** Baseline demographic and clinicopathologic characteristics of younger and elderly patients with EC

Variables	Age < 50 years old (*n* = 4415)		Age ≥ 50 years old (*n* = 36,267)		*P* value
	cases	%	cases	%	
Race					< 0.001
White	3310	75.0	29,535	81.5	
Black	333	7.5	3451	9.5	
Other	772	17.5	3281	9.0	
Grade					< 0.001
I	2466	55.9	14,351	39.6	
II	1211	27.4	9980	27.5	
III	583	13.2	8436	23.3	
IV	155	3.5	3500	9.6	
T stage					0.018
T1	3600	81.5	29,081	80.2	
T2	324	7.3	2580	7.1	
T3	441	10.1	4093	11.3	
T4	50	1.1	513	1.4	
N stage					< 0.001
N0	4023	91.1	31,958	88.1	
N1	237	5.4	2526	7.0	
N2	155	3.5	1783	4.9	
M stage					0.002
M0	4232	95.9	34,359	94.7	
M1	183	4.1	1908	5.3	
Tumor size (cm)					< 0.001
< 3.6	1116	25.3	7815	21.5	
3.6–7.8	3027	68.5	25,998	71.7	
> 7.8	272	6.2	2454	6.8	
SEER stage					< 0.001
Localized	3232	73.2	25,104	69.2	
Regional	978	22.2	9027	24.9	
Distant	205	4.6	2136	5.9	
Surgery					0.746
No	70	1.6	552	1.5	
Yes	4345	98.4	35,715	98.5	
Lymphadenectomy					< 0.001
No	1879	42.6	11,541	31.8	
Yes	2536	57.4	24,726	68.2	
Adjuvant treatment					< 0.001
No/Unknown	3111	70.5	21,493	59.3	
Only radiotherapy	435	9.9	6109	16.8	
Only chemotherapy	398	9.0	4198	11.6	
Chemoradiotherapy	471	10.6	4467	12.3	

*Abbreviations: Grade I* Well differentiated, *Grade II* Moderately differentiated, *Grade III/IV* Poorly differentiated or undifferentiated

Among EOEC patients, 70 patients were excluded from the study for not undergoing surgery, while 4,345 postoperative patients remained for further analysis. 3,044 patients were assigned into a training cohort and the remaining patients (*n* = 1,301) were assigned to an internal validation cohort. There were no statistically significant differences between the datasets and their baseline characteristics were summarized in Table 2.

**Table 2:** Baseline characteristics of the training set and the validation set

Variables	Whole population [cases (%)]	Training cohort [cases (%)]	Validation cohort [cases (%)]	*P*-value
Total	4345	3044	1301	
Age (years)				0.489
< 45	2290 (52.7)	1621 (53.2)	669 (51.5)	
45–47	1074 (24.7)	739 (24.3)	335 (25.7)	
47–49	981 (22.6)	684 (22.5)	297 (22.8)	
Race				0.116
White	3268 (75.2)	2263 (74.4)	1005 (77.2)	
Black	324 (7.5)	232 (7.6)	92 (7.1)	
Other	753 (17.3)	549 (18.0)	204 (15.7)	
Grade				0.141
I	2428 (55.9)	1690 (55.5)	738 (56.7)	
II	1202 (27.7)	836 (27.5)	366 (28.1)	
III	567 (13.0)	402 (13.2)	165 (12.7)	
IV	148 (3.4)	116 (3.8)	32 (2.5)	
T stage				0.749
T1	3555 (81.8)	2488 (81.7)	1067 (82.0)	
T2	313 (7.2)	216 (7.1)	97 (7.5)	
T3	430 (9.9)	309 (10.2)	121 (9.3)	
T4	47 (1.1)	31 (1.0)	16 (1.2)	
N stage				0.376
N0	3968 (91.4)	2768 (90.9)	1200 (92.2)	
N1	232 (5.3)	170 (5.6)	62 (4.8)	
N2	145 (3.3)	106 (3.5)	39 (3.0)	
M stage				0.326
M0	4172 (96.0)	2917 (95.8)	1255 (96.5)	
M1	173 (4.0)	127 (4.2)	46 (3.5)	
Tumor size (cm)				0.114
< 3.6	1098 (25.3)	750 (24.6)	348 (26.7)	
3.6–7.8	2979 (68.5)	2094 (68.8)	885 (68.0)	
> 7.8	268 (6.2)	200 (6.6)	68 (5.3)	
SEER stage				0.591
Localized	3196 (73.6)	2228 (73.2)	968 (74.4)	
Regional	954 (22.0)	674 (22.1)	280 (21.5)	
Distant	195 (4.4)	142 (4.7)	53 (4.1)	
Lymphadenectomy				0.584
No	1813 (41.7)	1262 (41.5)	551 (42.4)	
Yes	2532 (58.3)	1782 (58.5)	750 (57.6)	
Adjuvant treatment				0.353
No/Unknown	3070 (70.7)	2127 (69.9)	943 (72.5)	
Only radiotherapy	431 (9.9)	306 (10.1)	125 (9.6)	
Only chemotherapy	389 (9.0)	281 (9.2)	108 (8.3)	
Chemoradiotherapy	445 (10.4)	330 (10.8)	125 (9.6)	

*Abbreviations: Grade I* Well differentiated, *Grade II* Moderately differentiated, *Grade III/IV* Poorly differentiated or undifferentiated

### Establishment of the nomogram

**Fig. 3:**
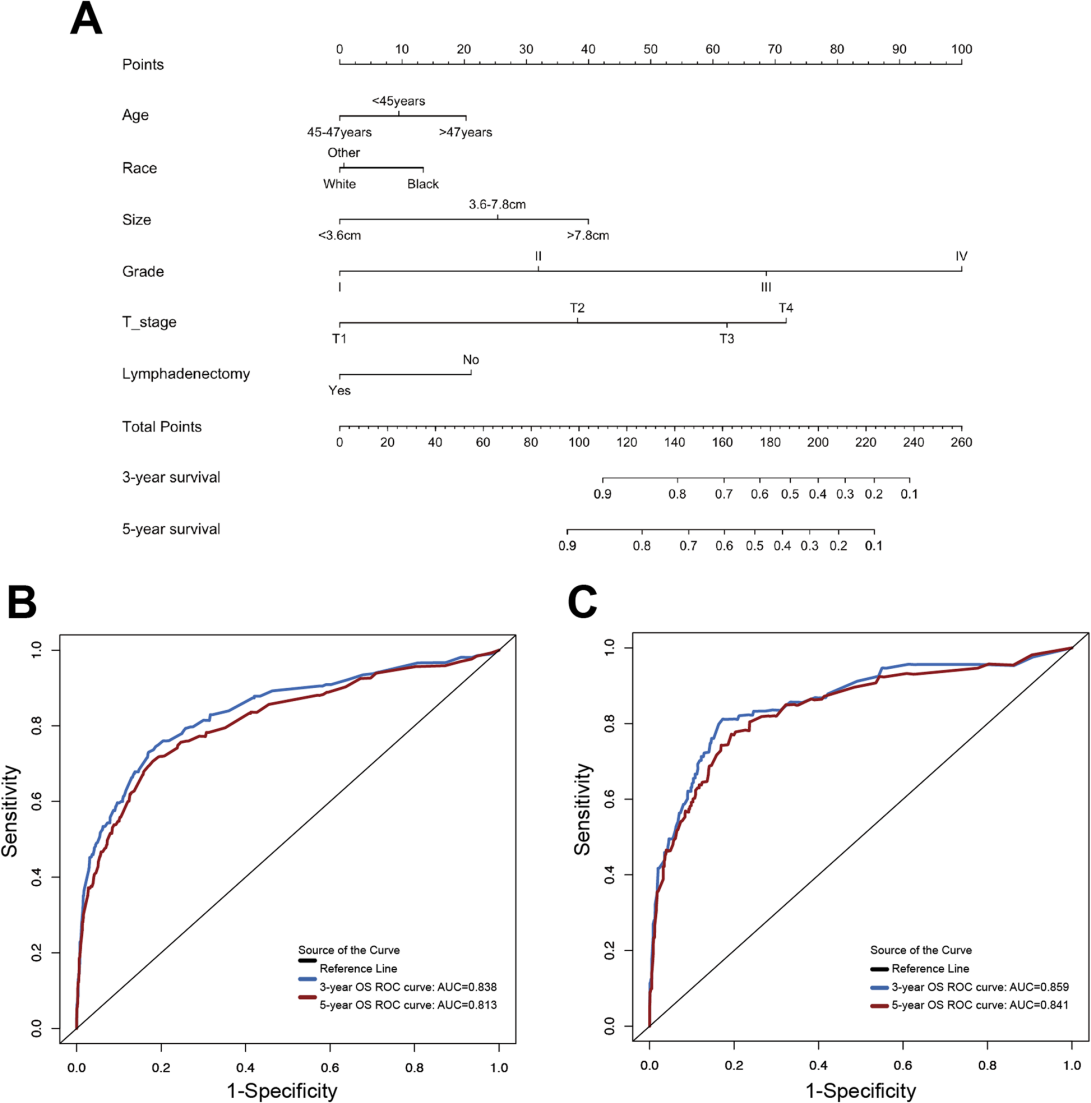
Nomograms for early-onset endometrial cancer patients predicting 3- and 5-year OS (**A**); ROC analysis of the nomogram model for 3- and 5-year OS in the training cohort (**B**); and the internal validation cohort (**C**)

Given that our study endpoint inherently involves time-to-event data, we performed univariate and multivariate Cox regression analyses to identify independent prognostic factors associated with OS (Table 3). Then, six predictors identified by the multivariate regression model were further recruited and used for developing OS nomogram model, including age [hazard ratio (HR) 1.375, 95% confidence interval (CI) 1.042–1.815], race (HR 1.547, 95% CI 1.096–2.183), grade (HR 8.903, 95% CI 5.665–13.993), T stage (HR 2.331, 95% CI 1.467–3.704), tumor size (HR 2.401, 95% CI 1.450–3.976), and lymphadenectomy (HR 0.698, 95% CI 0.526–0.927) (All *P* < 0.05, Fig. 3A). According to the degree of contribution of each predictor to the resulting events (OS), the corresponding points (the first axis) were obtained. Then, the points of each predictor were summed to predict the survival probability of EOEC patients.

**Table 3:** Univariate and multivariate analyses for OS in the training cohort (*n* = 3044)

Variables	Univariate analysis			Multivariate		
	HR	95% CI	*P* value	HR	95% CI	*P* value
Age (years)						
< 45	1.000	-	0.003	1.000	-	0.019
45–47	0.976	0.719–1.325	0.878	0.883	0.646–1.208	0.437
47–49	1.548	1.179–2.032	0.002	1.375	1.042–1.815	0.025
Race						
White	1.000	-	< 0.001	1.000	-	0.045
Black	2.373	1.701–3.311	< 0.001	1.547	1.096–2.183	0.013
Other	1.282	0.950–1.732	0.104	1.124	0.826–1.529	0.457
Grade						
I	1.000	-	< 0.001	1.000	-	< 0.001
II	2.714	1.907–3.863	< 0.001	2.029	1.402–2.937	< 0.001
III	8.795	6.316–12.247	< 0.001	4.231	2.873–6.233	< 0.001
IV	19.427	13.238–28.511	< 0.001	8.903	5.665–13.993	< 0.001
T stage						
T1	1.000	-	< 0.001	1.000	-	0.005
T2	4.100	2.851–5.896	< 0.001	1.899	1.142–3.157	0.013
T3	9.241	7.108–12.014	< 0.001	2.331	1.467–3.704	< 0.001
T4	15.415	8.839–26.884	< 0.001	1.951	0.857–4.443	0.111
N stage						
N0	1.000	-	< 0.001	1.000	-	0.361
N1	4.399	3.199–6.050	< 0.001	1.258	0.870–1.820	0.222
N2	5.636	3.931–8.079	< 0.001	0.943	0.621–1.432	0.783
M stage						
M0	1.000	-	< 0.001	1.000	-	-
M1	13.686	10.480–17.873	< 0.001	1.897	0.633–5.680	0.253
Tumor size (cm)						
< 3.6	1.000	-	< 0.001	1.000	-	0.002
3.6–7.8	2.783	1.876–4.130	< 0.001	1.960	1.304–2.947	0.001
> 7.8	6.148	3.802–9.942	< 0.001	2.401	1.450–3.976	0.001
SEER stage						
Localized	1.000	-	< 0.001	1.000	-	0.356
Regional	4.023	3.040–5.323	< 0.001	1.331	0.821–2.157	0.246
Distant	21.766	16.091–29.442	< 0.001	2.097	0.627–7.012	0.229
Lymphadenectomy						
No	1.000	-	0.009	1.000	-	-
Yes	1.387	1.081–1.780	0.010	0.698	0.526–0.927	0.013
Adjuvant treatment						
No/Unknown	1.000	-	< 0.001	1.000	-	0.137
Only radiotherapy	2.508	1.677–3.749	< 0.001	1.041	0.667–1.624	0.859
Only chemotherapy	9.465	7.108–12.602	< 0.001	1.231	0.815–1.861	0.323
Chemoradiotherapy	4.309	3.096–5.996	< 0.001	0.808	0.521–1.253	0.341

*Abbreviations: HR* Hazard ratio, *CI* Confidence intervals, *Grade I* Well differentiated, *Grade II* Moderately differentiated, *Grade III/IV* Poorly differentiated or undifferentiated

### Internal validation of the nomogram

**Fig. 4:**
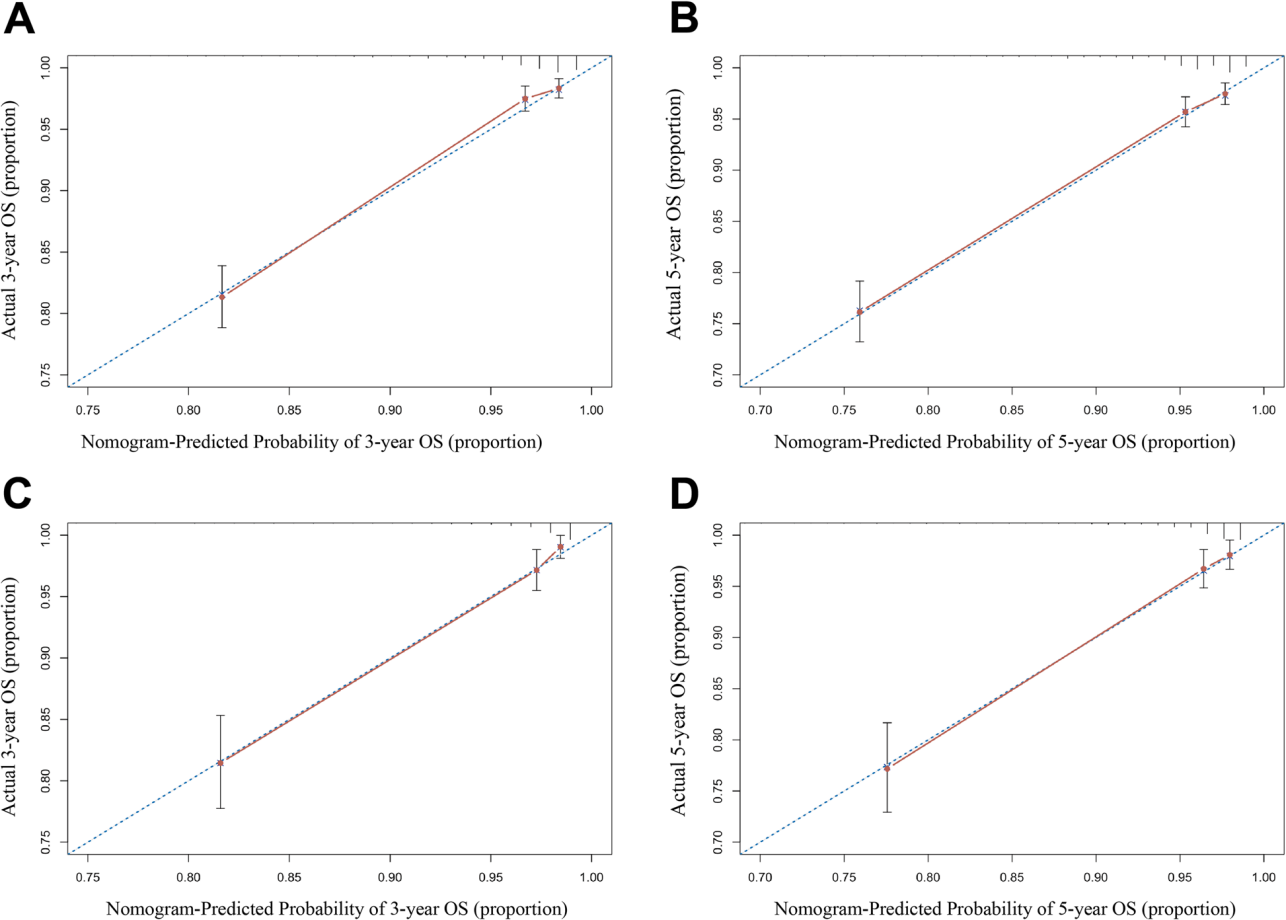
Calibration plots of nomograms for 3- and 5-year OS in the training cohort (**A**, **B**); the internal validation cohort (**C**, **D**)

The Harrell’s C-index measures a model's predictive ability for survival time, while the ROC curve and its area under the curve (AUC) evaluate the model's classification performance at various thresholds. Values closer to 1 for both indicate superior predictive performance of the model [22–24]. The established nomogram model was further verified with C-index and AUC values. In the OS prediction model, the C-index values for the training cohort were 0.816 (95% CI: 0.787–0.845). For the internal validation cohort, the C-index values were 0.841 (95% CI: 0.798–0.884). AUC values of the models were above 0.8, demonstrating that the models had good discriminatory ability (Fig. 3B and C). The calibration curves demonstrated that there were only minor deviations from perfect consistency between the predicted and observed values, implying that the nomogram model fit well (Fig. 4).

**Fig. 5:**
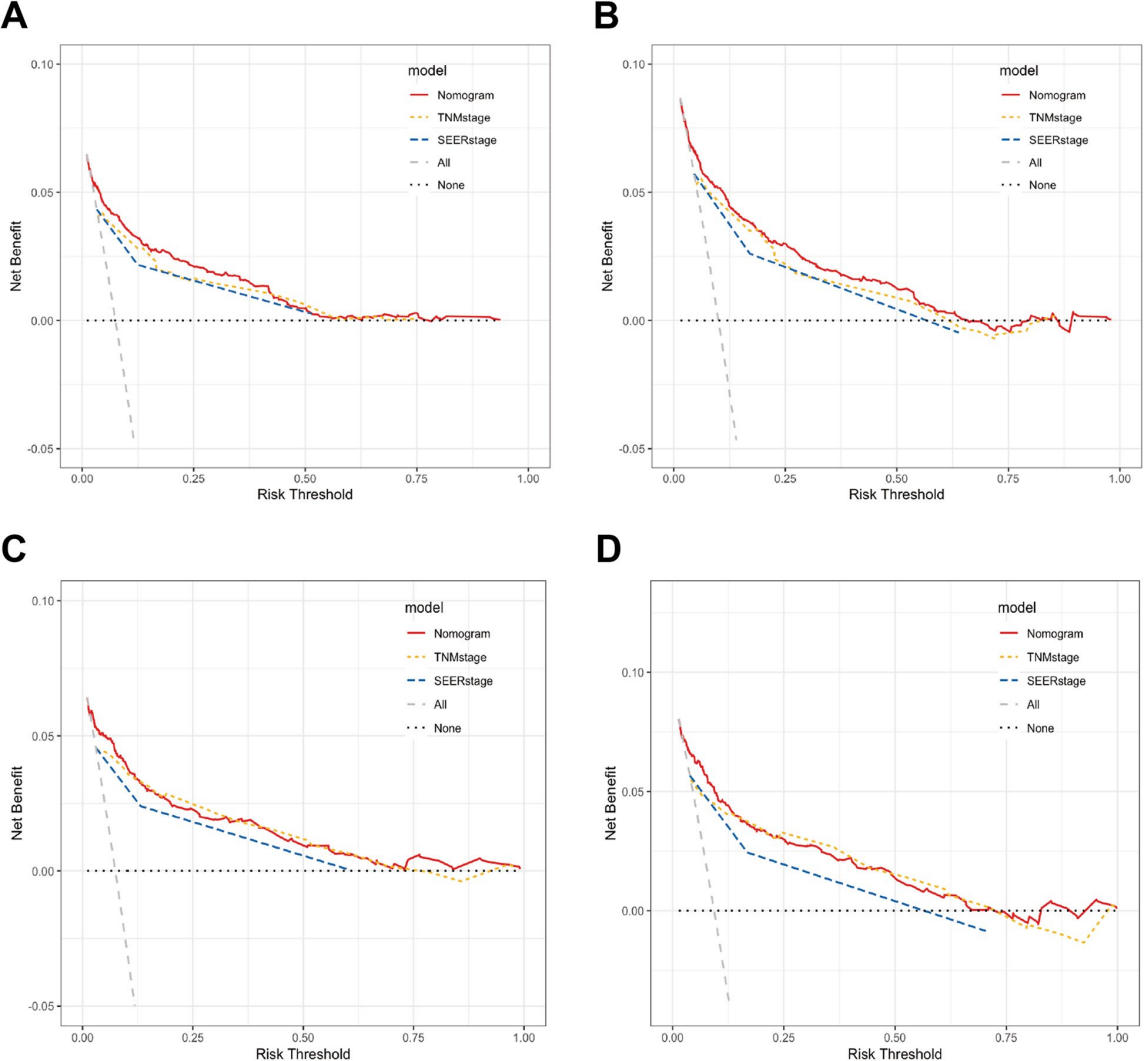
DCA for different risk stratification systems for 3- and 5-year OS in the training cohort (**A**, **B**); the internal validation cohort (**C**, **D**)

In addition, we evaluated the merits and demerits of the newly constructed nomogram by comparing with TNM stage and SEER stage. First, we used C-index to verify the prediction model for OS, which was better than the TNM stage and SEER stage (Table 4). Moreover, the DCA results also implied that the clinical applicability of the model was superior to TNM stage and SEER stage (Fig. 5).

**Table 4:** C-index of different risk stratification systems for OS in the training and validation set

Risk stratification systems	Training set		Internal validation set		External validation set	
	C-index	95% CI	C-index	95% CI	C-index	95% CI
AJCC TNM stage	0.765	(0.736–0.794)	0.780	(0.733–0.827)	0.730	(0.509–0.951)
SEER stage	0.753	(0.723–0.782)	0.772	(0.727–0.817)	0.696	(0.502–0.890)
Nomogram model	0.816	(0.787–0.845)	0.841	(0.798–0.884)	0.898	(0.806–0.991)

*Abbreviations: C-index* Concordance index, *CI* Confidence interval, *OS* Overall survival, *AJCC TNM* Stage, tumor size/nodes/metastasis, *SEER* Stage, classified as local, regional, and distant based on the localized site or extent of cancer

### External validation of the nomogram

**Fig. 6:**
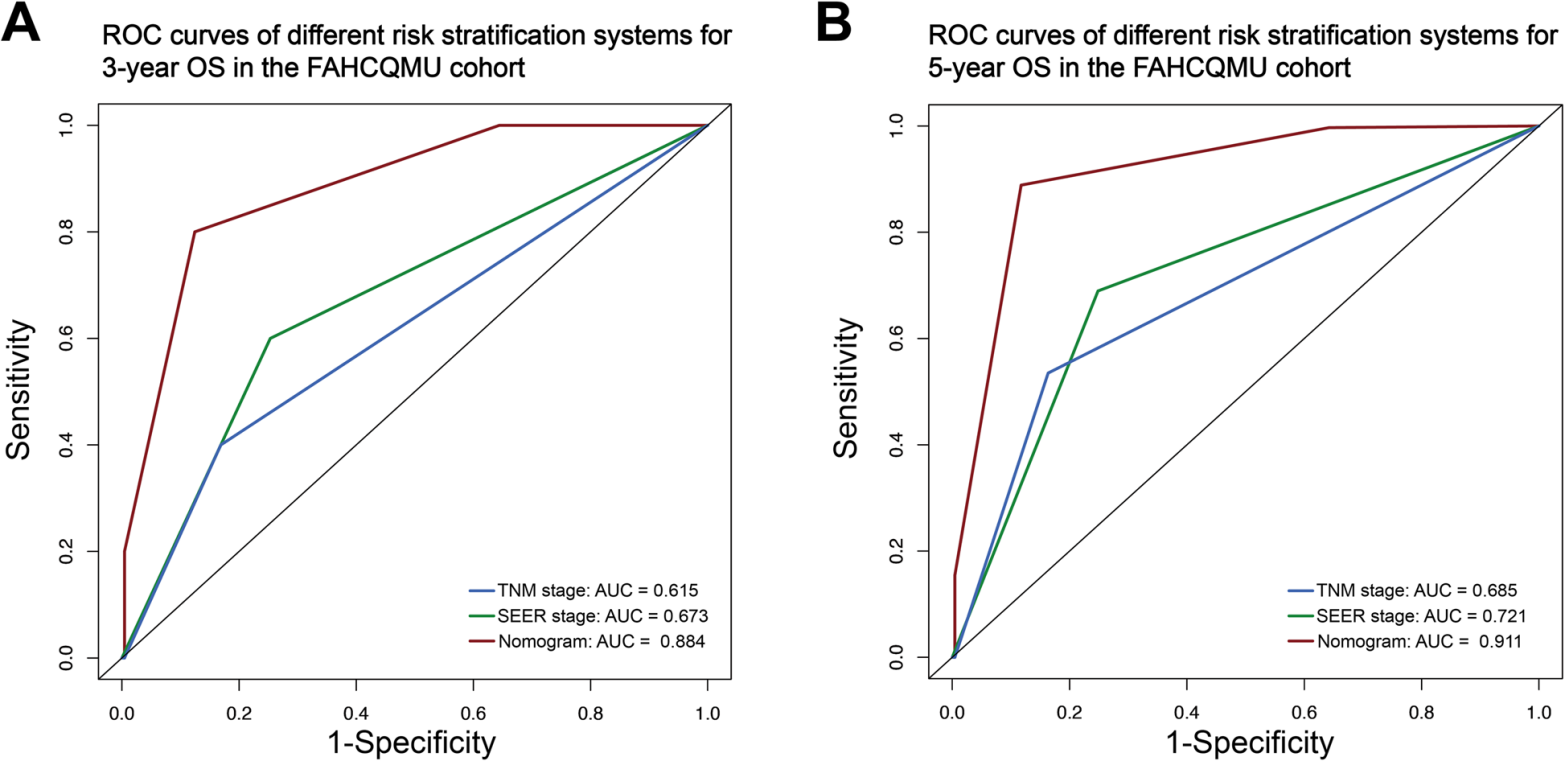
ROC analysis of different risk stratification systems for 3-year OS in the external validation cohort (**A**); for 5-year OS in the external validation cohort (**B**)

To further evaluate the performance of the proposed nomogram model, 230 patients with EOEC who received primary surgical treatment at the First Affiliated Hospital of Chongqing Medical University were involved in this study for external validation. The ages of patients and tumor sizes in the external validation data were classified according to the optimal cutoff values established from the SEER database. Among the EOEC patients, there were 85 cases (36.9%) of those aged 45 to 47 years, while there were 58 cases (25.3%) of those aged 47 to 50 years (excluding 50 years). Among the patients aged 47 to 49 years, the majority were perimenopausal women, who frequently presented with menstrual disorders at the time of diagnosis. The majority of patients were classified as T stage 1 and 2, accounting for 99.6% of the entire cohort. There were 81 (35.2%), 119 (51.7%), and 30 (13.1%) patients in Grade I (well differentiated), Grade II (moderately differentiated), and Grade III (poorly differentiated). In terms of treatment, a considerable proportion of patients in this cohort underwent lymph node dissection (97.8%, 225 out of 230 cases). During the follow-up period, 6 (2.6%) patients died, of which 5 (2.2%) patients died due to EC. Detailed information on the clinical and pathological features is listed in Supplementary Table 2. The external dataset is stored on [Harvard Dataverse] at [https://doi.org/10.7910/DVN/Z7C1HC] and is openly available [25]. As for the external validation cohort, the C-index values were 0.898 (95% CI: 0.806–0.991). The AUC values of the nomogram for FAHCQMU cohort at 3- and 5-year OS were 0.884 and 0.911, which were higher than the TNM stage (0.615 and 0.685) and SEER stage (0.673 and 0.721) (Fig. 6).

### Survival analysis

**Fig. 7:**
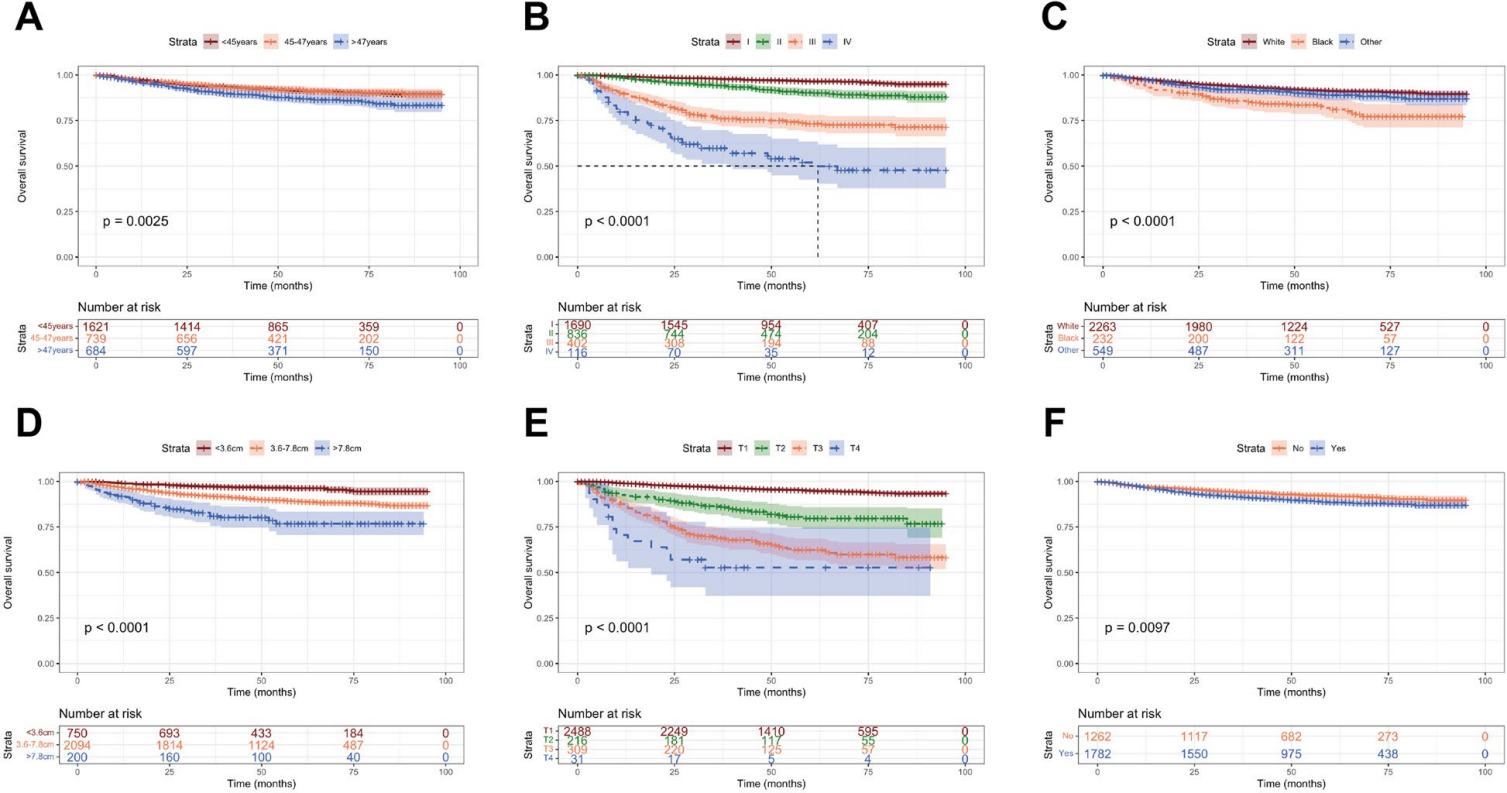
Survival analysis to determine the impact of (**A**) age; (**B**) tumor size; (**C**) race; (**D**) T stage; (**E**) grade; and (**F**) lymphadenectomy on OS

The Kaplan–Meier method was used to evaluate the OS among EOEC patients (Fig. 7). Age had an impact on survival, with patients aged > 47 years seeming to have a worse prognosis. Black patients had the shorter survival time than other races, and the prognosis of patients with tumors > 7.8 cm seems to have a worse survival. In terms of tumor characteristics, T stage and tumor grade were respectively related to the outcome of EOEC patients. Patients with T4 stage or grade IV had worse OS significantly. Meanwhile, OS improved with lymphadenectomy compared to untreated patients.

**Fig. 8:**
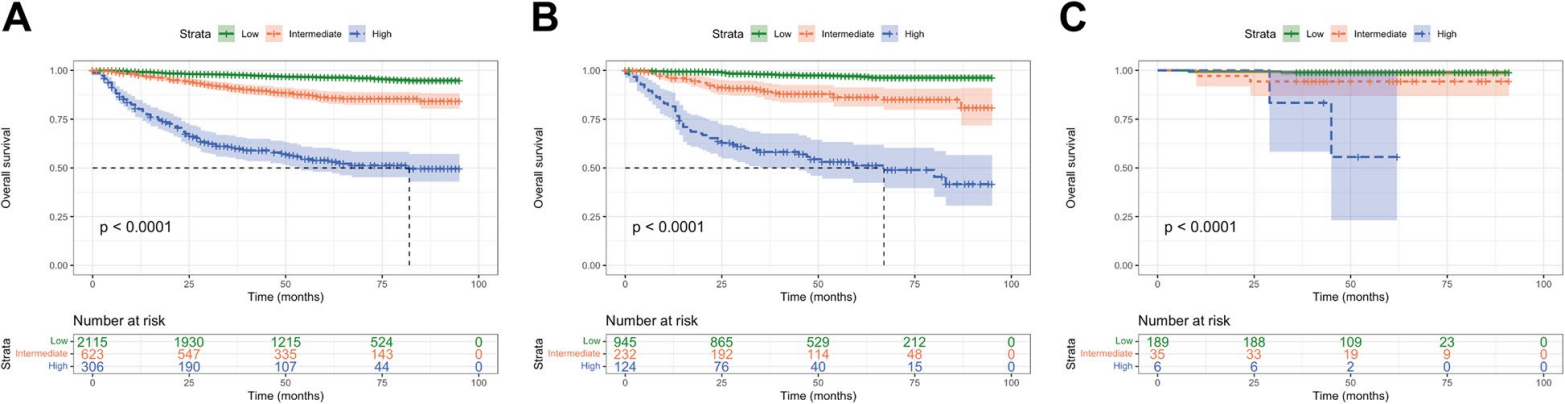
Overall survival of EOEC patients stratified by risk in the training set (**A**), the internal validation set (**B**), and the external validation set (**C**)

To further validate the risk stratification ability of the predictive model, we categorized EOEC cases into high-risk group (total score > 118.4 pts), intermediate-risk group (total score: 71.1–118.4 pts), and low-risk group (total score < 71.1 pts) based on the nomogram-generated scores (Supplementary Fig. 3). Kaplan–Meier survival curves indicated that patients in the high-risk group had the worst survival outcomes; whereas those in the low-risk group had the best overall survival (*P* < 0.001; Fig. 8).

## Discussion

The overall incidence of EC has increased in recent years, especially among younger patients. EOEC has gradually become a unique subset due to the significant differences in clinical manifestations, clinicopathological features, and survival prognosis [26]. In our present study, although young patients with EC accounted for only 10.9%, due to the large population base, this represents a substantial and increasing number [10]. Thus, it is of great significance to accurately predict the survival time of EOEC patients by comprehensively considering multiple clinical features. Here, we constructed and validated a specialized survival nomogram to predict individual postoperative survival outcomes in EOEC.

Surgery is the best approach to treat EC; however, in cases of EOEC, a notable portion of patients choose conservative treatment to preserve fertility. Nevertheless, these patients often experience a higher risk of tumor relapse [27]. In the study by Son J et al. [28], 96.1% of young patients with EC underwent hysterectomy, whereas patients who opted for fertility-sparing treatment with progestin therapy were associated with higher recurrence rates. Therefore, surgical treatment is crucial for improving the prognosis and survival of patients with EOEC. Our preliminary research has also confirmed this. Notably, a recent study by Bogani et al. on “oldest old” EC patients demonstrated that minimally invasive surgery (i.e., vaginal, laparoscopic, and robotic-assisted surgery) is both safe and effective for treating patients aged 85 years and older. The study indicated that the choice of surgical approach did not significantly affect disease-free survival and overall survival rates, emphasizing that age should not be regarded as a contraindication for surgical intervention. This finding further underscores the importance of surgical treatment across diverse age groups of EC patients. It is advisable to conduct independent studies focusing on the cohort receiving fertility-sparing treatment. Additionally, the substantial data gaps for non-surgical patients in the SEER database could introduce selection bias underscoring the need for a study that primarily examines this subset of young women undergoing surgical treatment and assesses their risk stratification.

In this study, ten factors of demographic and clinical characteristics were analyzed, and six factors were determined as predictors for constructing nomogram, which consisted of age, tumor size, race, grade, T stage, and lymphadenectomy. These variables have been reported to be associated with the prognosis of EOEC patients [29]. In this study, age was an independent prognostic factor for EOEC patients. Elderly patients with EC are often accompanied by some basic diseases, such as diabetes, hypertension, neurological diseases, etc., which cause deterioration of patients’ condition. For young patients, their physical fitness, immunity, and tolerance are relatively good, allowing for earlier diagnosis and a better prognosis. More interestingly, we observed a higher risk of death in patients aged between 47 and 49 years; similarly with multivariate analysis results, survival analysis also indicated a worse prognosis for patients in this age group. It is speculated that some premenopausal women aged between 47 and 49 years mostly presented clinically with menstrual disorders that cover up EOEC and delay the diagnosis, which may be a potential cause of adverse prognosis. Therefore, clinicians should be alert to these women with risk factors and treat them aggressively; conversely, patients younger than 47 years should be evaluated thoroughly to avoid overtreatment.

A number of studies have demonstrated race is associated with the prognosis of EC, which were consistent with our findings [30]. Our present study suggested that black women with EOEC had a worse prognosis compared to white women, which may be related to changes in people’s lifestyle and eating habits in recent years. An epidemiological report indicated that although the incidence of EC in black women is similar to that of whites, the death rate is 97% higher than that in white women [31]. The difference in cancer prognosis can be attributed in part to genetic differences, the majority of race disparity is driven by variations in socioeconomic status and access to quality medical care [30, 32]. Therefore, it is crucial to support black women in accessing healthcare services, enhancing the quality of treatment, reducing incidence rates, and improving their overall quality of life. By measuring the weights of variables on the nomogram scale, we found that tumor grade was the most important prognostic factor for EOEC, with a higher grade frequently indicating a worse prognosis. Despite the overall survival rate of patients being relatively high, high-grade EC tend to recur, which is one of the main causes of death [33]. In AJCC and FIGO staging, the T staging of EC is primarily based on whether the tumor is confined to the uterine body and its extent of invasion. However, tumor size has not yet been incorporated into the staging system for EC, despite its widespread use in risk assessment and treatment decision-making for various cancers, including cervical and breast cancer [16]. Our present study demonstrated that tumor size and T stage were closely related to the survival status of patients with EOEC, especially when the tumor size is larger than 7.8 cm (HR 2.401, 95% CI 1.450–3.976) and in cases of deep tumor invasion (T3: HR 2.331, 95% CI 1.467–3.704), which is associated with worse OS. Accumulating evidence has confirmed that tumor size is closely related to the grading and staging of EC [34, 35]. Notably, tumor size is considered a negative predictive factor in EC [36]. A study based on the SEER database included 52,208 patients with all-stage of EC who underwent total hysterectomy. The results indicated that tumor size, as a continuous variable, has significant prognostic predictive value, with an optimal cutoff value of 3.9 cm. As tumor size increases, the risk of mortality for patients also rises, demonstrating a nonlinear relationship between the two. Initially, the risk of mortality increased rapidly with tumor size, but when the tumor diameter exceeds 7.5 cm, the rate of risk increase slows down. When tumor size was evaluated as a categorical variable, the highest mortality risk was observed in patients with tumors larger than 9 cm, showing an HR of 3.17 in univariate Cox analysis (95% CI 2.87–3.51, *P* < 0.001) and an HR of 1.61 in multivariate Cox analysis (95% CI 1.46–1.79, *P* < 0.05) [37]. Research by Chattopadhyay et al. has demonstrated that tumor size is a significant assessment factor in risk stratification of EC. For women with tumors larger than 3.75 cm, the five-year cancer-related survival rate is 55%, compared to 75% for those with tumors measuring 3.75 cm or less. Our study further confirms that for patients with EOEC, the risk of mortality increases when the tumor diameter is between 3.6 and 7.8 cm; additionally, when the tumor diameter exceeds 7.8 cm, the OS rate declines significantly. Therefore, clinicians should remain vigilant for EOEC patients with tumor diameters exceeding 3.6 cm. Lymph node metastasis is the most crucial prognostic factor in EC, and effective lymph node dissection can significantly improve the prognosis of EC patients. On one hand, lymph node dissection can provide valuable information for guiding postoperative adjuvant therapies. On the other hand, by eradicating metastatic disease and reducing tumor burden, lymphadenectomy can provide therapeutic benefits to patients. However, the application of lymph node dissection in early-stage EC still lacks randomized clinical research evidence. Our study indicates that lymphadenectomy is an independent prognostic factor in EOEC, and effective lymph node dissection can prolong the OS of EC. The study by Havrilesky et al.[38] demonstrated that patients with lymph node metastasis who underwent complete resection of lymph node metastasis had significantly higher 5-year disease-specific survival compared to those with residual lymph node metastasis (unresectable nodal disease or macroscopic metastasis) at surgery completion. These findings support the aggressive resection of evident lymph node metastasis [39]. Another study by Bendifallah et al. [40] indicated no significant difference in survival between patients with or without lymphadenectomy for any grade after matching, except for grade 3 cancers. Generally, there is still widespread debate regarding the therapeutic benefits of lymph node dissection, especially for younger patients with EC. While lymph node dissection can provide more accurate staging, the actual therapeutic effects of this procedure still require further confirmation through prospective studies. Additionally, the potential of sentinel lymph node assessment to replace complete lymph node dissection for identifying lymph node metastasis in EOEC patients is also worth further exploration.

Notably, N stage, M stage, radiotherapy, and chemotherapy were not candidate predictors for EOEC in the present study. Actually, N and M stages were considered as essential risk factors for EC patients, we considered that these disagreements arose from our focus on EOEC patients rather than all EC patients, and some high-risk clinical features, including high N and M stages, were infrequently found in EOEC. Hence, using these factors to predict the survival of EOEC may not be reliable. More intriguingly, younger patients were less likely to receive adjuvant therapy than older groups, making radiotherapy and chemotherapy not significant in multivariate analysis [28].

In recent years, many predictive models and molecular classifications have been established to predict the survival status of EC patients. However, the high cost of testing and the cumbersome procedures make it difficult to carry out in many regions. The nomogram we established was based on six readily available clinical characteristics, which are more beneficial to clinicians in assessing patients’ prognosis and making appropriate clinical decisions. For instance, a 49-year-old Chinese woman (20 pts) was diagnosed with EC postoperatively. During the surgery, lymphadenectomy was not performed (20 pts), the tumor size measured 4 cm (24 pts), the tumor differentiation was classified as poorly differentiated/Grade III (69 pts), and the T stage was II (38 pts). According to the nomogram, this patient had a total score of 173 pts, categorizing her into the high-risk group (exceeding the model’s threshold of 118.4 pts). Consequently, enhanced monitoring and management, including quarterly follow-ups and imaging examinations, are essential for her care. This tool undoubtedly provides a quick and convenient approach for personalized risk assessment. Additionally, our model was based on a large, representative population-based dataset from SEER and further validated using an independent cohort of Chinese EOEC patients, which enhances its generalizability. Moreover, the analysis of only EOEC cases provided an opportunity to comprehensively consider the variables incorporated into the model. The nomogram integrating multiple clinical variables outperformed the TNM stage and SEER stage, showing good prognostic discrimination in patients with EOEC. It is important to clarify that, regarding clinical characteristics, 86.9% of patients in the FAHCQMU cohort were classified as Grade I-II, while 13.1% were classified as Grade III-IV. In comparison, the SEER cohort had proportions of 83.6% and 16.4%, respectively, with a similar age distribution. However, the population included in this study consists entirely of Chinese patients, and the majority of whom underwent lymphadenectomy postoperatively. This difference may contribute to variations in the model's performance between the two cohorts (Supplementary Table 3). In terms of clinical outcomes, the predictive model effectively stratified the risk for EOEC patients across different cohorts. In the FAHCQMU cohort, the 3-year and 5-year OS rates for high-risk patients were 83.3% (53.5%-100.0%) and 55.6% (26.2%-85.0%), respectively. In contrast, the training set of the SEER cohort exhibited 3-year and 5-year OS rates of 60.7% (55.0%-66.4%) and 53.8% (47.7%-59.9%), while the validation set showed 3-year and 5-year OS rates of 58.1% (49.3%-66.9%) and 51.2% (41.6%-60.8%) (Supplementary Table 4). These results suggest that the predictive model maintains robust predictive capability across patient populations with different characteristics.

However, there are some limitations in our study. First, the retrospective nature of data collection from the SEER database introduces inherent biases, including potential inconsistencies in data recording, missing variables, and unmeasured confounders. These limitations may affect the reliability of our findings and their generalizability to broader populations. Another major limitation is the lack of detailed treatment records in the SEER database, such as specific surgical procedures, chemotherapy regimens, as well as targeted therapy and immunotherapy strategies. This omission precludes a comprehensive analysis of treatment-outcome relationships. Additionally, when performing survival analysis on the elderly and the young groups, the imbalance in sample sizes may impact statistical power and result in biased estimates of the survival curves. Secondly, the lack of data on lymphovascular invasion, histological subtypes, genomic data, and molecular profiling in the SEER database limits our ability to fully assess the prognostic impact of these factors on patients with EOEC. Therefore, the prognostic significance of these factors warrants further investigation in future studies and should be incorporated into predictive models. Furthermore, our study relied on retrospective data from the SEER cohort combined with a single-center cohort, which may introduce selection bias and limit the external validity of our results. The SEER database primarily represents the U.S. population; thus, differences in genetic backgrounds, lifestyle factors (e.g., diet, environmental exposures), and healthcare systems between Eastern and Western populations could influence global applicability of our model. To address these constraints, future research should utilize prospective international multicenter studies to validate our model across diverse ethnic and geographic populations. These efforts will enhance generalizability and reduce biases from retrospective data collection. Additionally, future studies should systematically collect standardized treatment documentation, including specific protocols, dose intensity, and treatment adherence, to elucidate the effects of different treatment strategies on survival outcomes in EOEC. Transparency regarding these limitations is essential for appropriately interpreting our results and guiding further investigations into the prognosis of EOEC.

## Conclusion

In summary, we developed a prognostic model for individualized survival prediction in EOEC patients. This tool is clinically accessible and enables effective risk stratification, thereby guiding personalized patient management.

## Supplementary Information

Supplementary Material 1. Supplementary Fig. 1. Flowchart for the inclusion and exclusion criteria of external validation dataset for EOEC patients (*n* = 230). Supplementary Fig. 2. The optimal thresholds for patients age and tumor size in the entire cohort were assessed by X-tile. Supplementary Table 1. Analysis of survival differences between non-surgical and surgical groups in EOEC cohort. Supplementary Table 2. Clinicopathological characteristics of the external validation cohort. Supplementary Table 3. Clinicopathological characteristics of the SEER cohort and the FAHCQMU cohort. Supplementary Table 4. Analysis of survival differences between high-, intermediate-, and low-risk groups in the SEER cohort and the FAHCQMU cohort. Supplementary Fig. 3. Cut-off values calculated by X-tile software (A) and (B). The determined cut-off value was 71.1 and 118.4, categorizing EOEC patients into high-risk group (total score > 118.4 pts), intermediate-risk group (total score: 71.1-118.4 pts), and low-risk group (total score < 71.1 pts).

Supplementary Material 2. Supplementary Materials R. Related computerized programs for nomogram with R.

Supplementary Material 3.

## Data Availability

The data that support the findings of this study are openly available in [Harvard Dataverse] at [10.7910/DVN/Z7C1HC]. Publicly available datasets were analyzed in this study. These data can be found in the SEER database (https://seer.cancer.gov/).

## Abbreviations

EOEC: Early-onset endometrial cancer
SEER: Surveillance, Epidemiology, and End Results
AJCC: American Joint Committee on Cancer
OS: Overall survival
ROC: Receiver operating characteristic
C-index: Concordance index
DCA: Decision curve analysis
HR: Hazard ratio
CI: Confidence interval
