# Supplementary figures and images for: Comparing survival outcomes between surgical and non-surgical treatments in patients with early-onset endometrial cancer and developing a nomogram to predict survival: a study based on Eastern and Western data sets
Source: World J Surg Oncol. 2025 May 11;23:184. doi: 10.1186/s12957-025-03825-y (PMC12067707; doi:10.1186/s12957-025-03825-y)

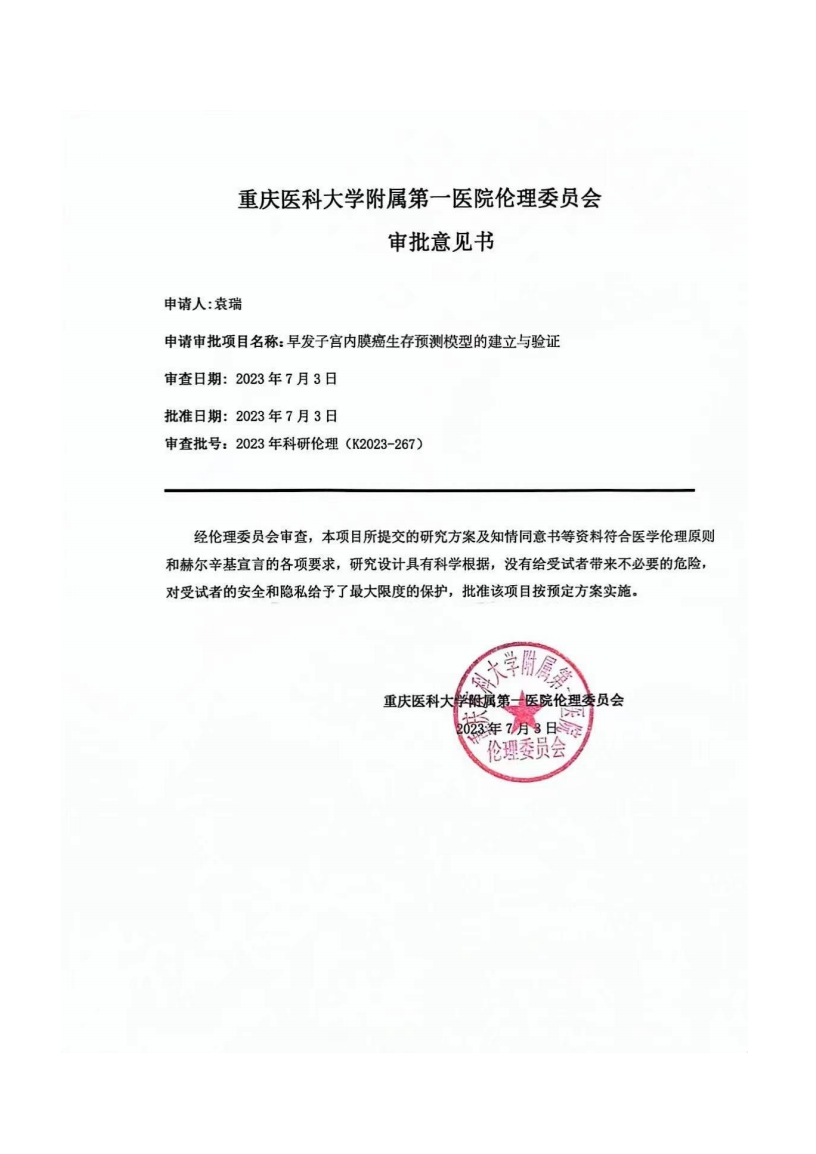


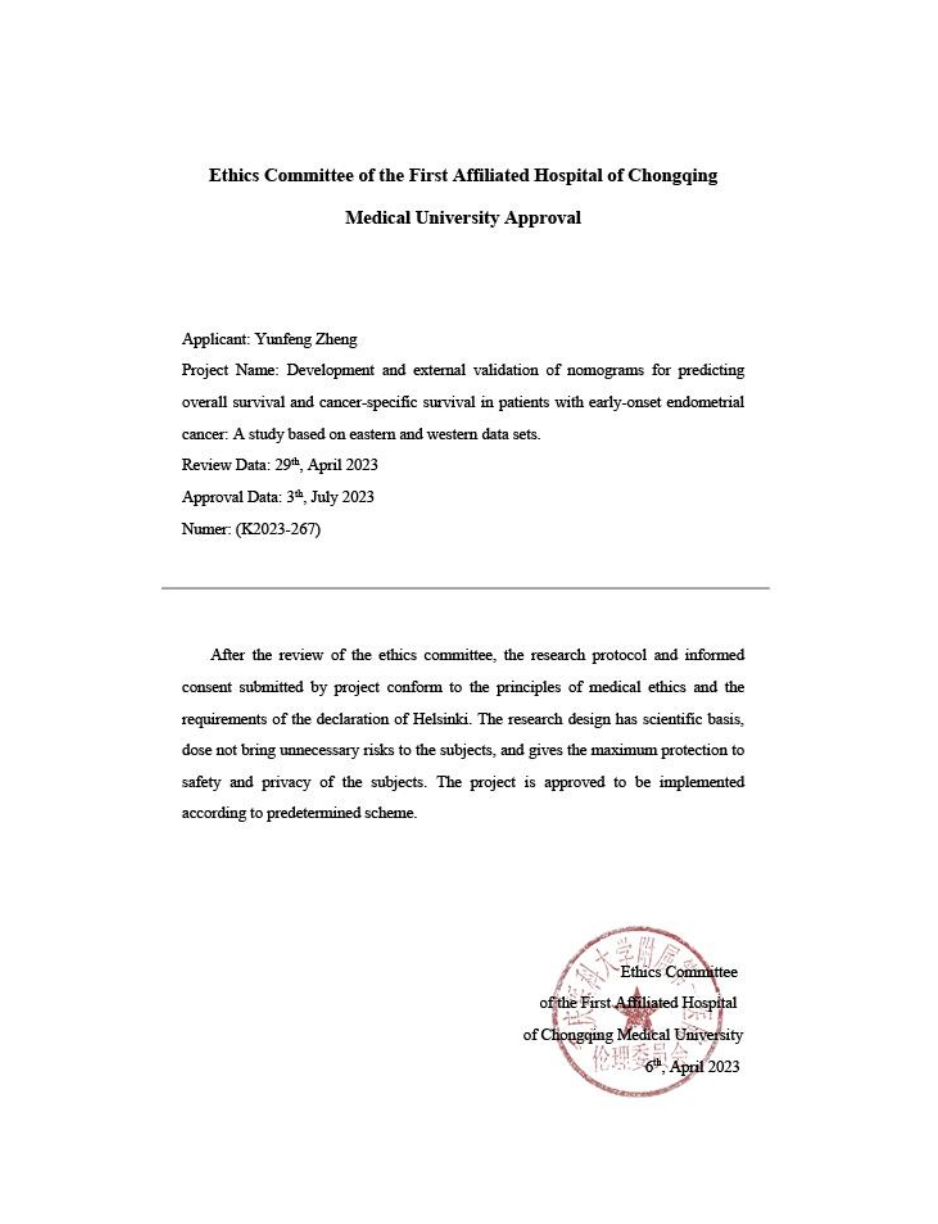

Supplement: Supplementary file 1 — Supplementary Material 1. Supplementary Fig. 1. Flowchart for the inclusion and exclusion criteria of external validation dataset for EOEC patients (n = 230). Supplementary Fig. 2. The optimal thresholds for patients age and tumor size in the entire cohort were assessed by X-tile. Supplementary Table 1. Analysis of survival differences between non-surgical and surgical groups in EOEC cohort. Supplementary Table 2. Clinicopathological characteristics of the external validation cohort. Supplementary Table 3. Clinicopathological characteristics of the SEER cohort and the FAHCQMU cohort. Supplementary Table 4. Analysis of survival differences between high-, intermediate-, and low-risk groups in the SEER cohort and the FAHCQMU cohort. Supplementary Fig. 3. Cut-off values calculated by X-tile software (A) and (B). The determined cut-off value was 71.1 and 118.4, categorizing EOEC patients into high-risk group (total score > 118.4 pts), intermediate-risk group (total score: 71.1-118.4 pts), and low-risk group (total score < 71.1 pts). [file 12957_2025_3825_MOESM1_ESM.docx]
