# Supplementary material for: Comparing survival outcomes between surgical and non-surgical treatments in patients with early-onset endometrial cancer and developing a nomogram to predict survival: a study based on Eastern and Western data sets
Source: World J Surg Oncol. 2025 May 11;23:184. doi: 10.1186/s12957-025-03825-y (PMC12067707; doi:10.1186/s12957-025-03825-y)
Supplement: Supplementary file 2 — Supplementary Material 2. Supplementary Materials R. Related computerized programs for nomogram with R. [file 12957_2025_3825_MOESM2_ESM.docx]

**Supporting Information**

**Comparing survival outcomes between surgical and non-surgical treatments in patients with early-onset endometrial cancer and developing a nomogram to predict survival: A study based on Eastern and Western data sets**

*Yunfeng Zheng, Ran Hu, Fan Yang, Gaohua Liu, Tianyu Peng, Langting Xie, Jie Wu, Lamei Hou, and Rui Yuan*


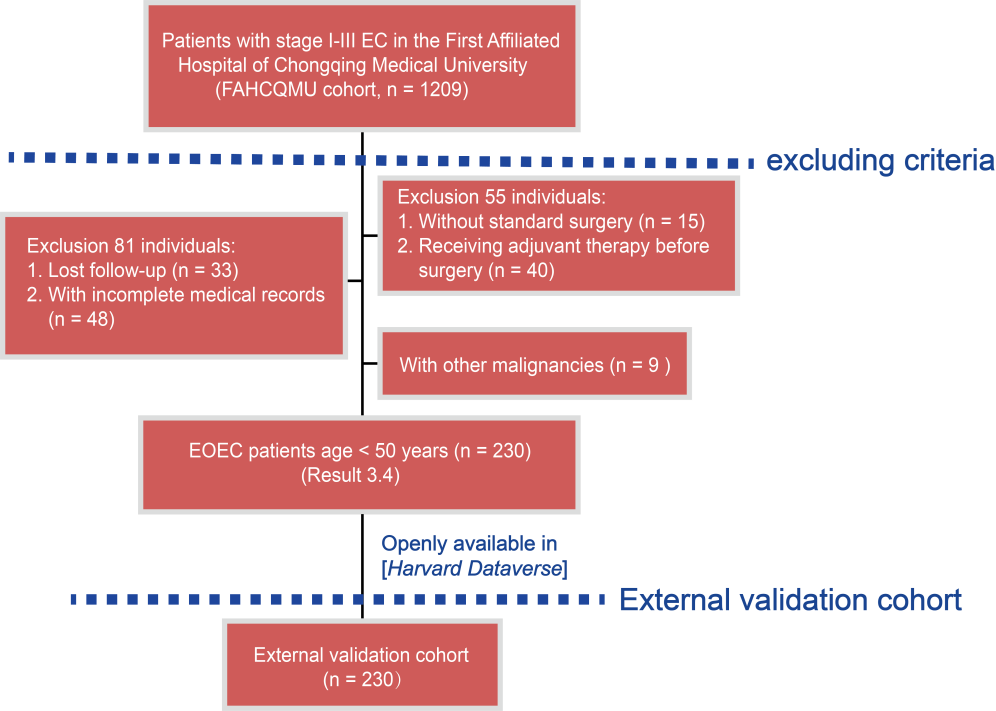


**Supplementary Fig. 1** Flowchart for the inclusion and exclusion criteria of external validation dataset for EOEC patients (n = 230).


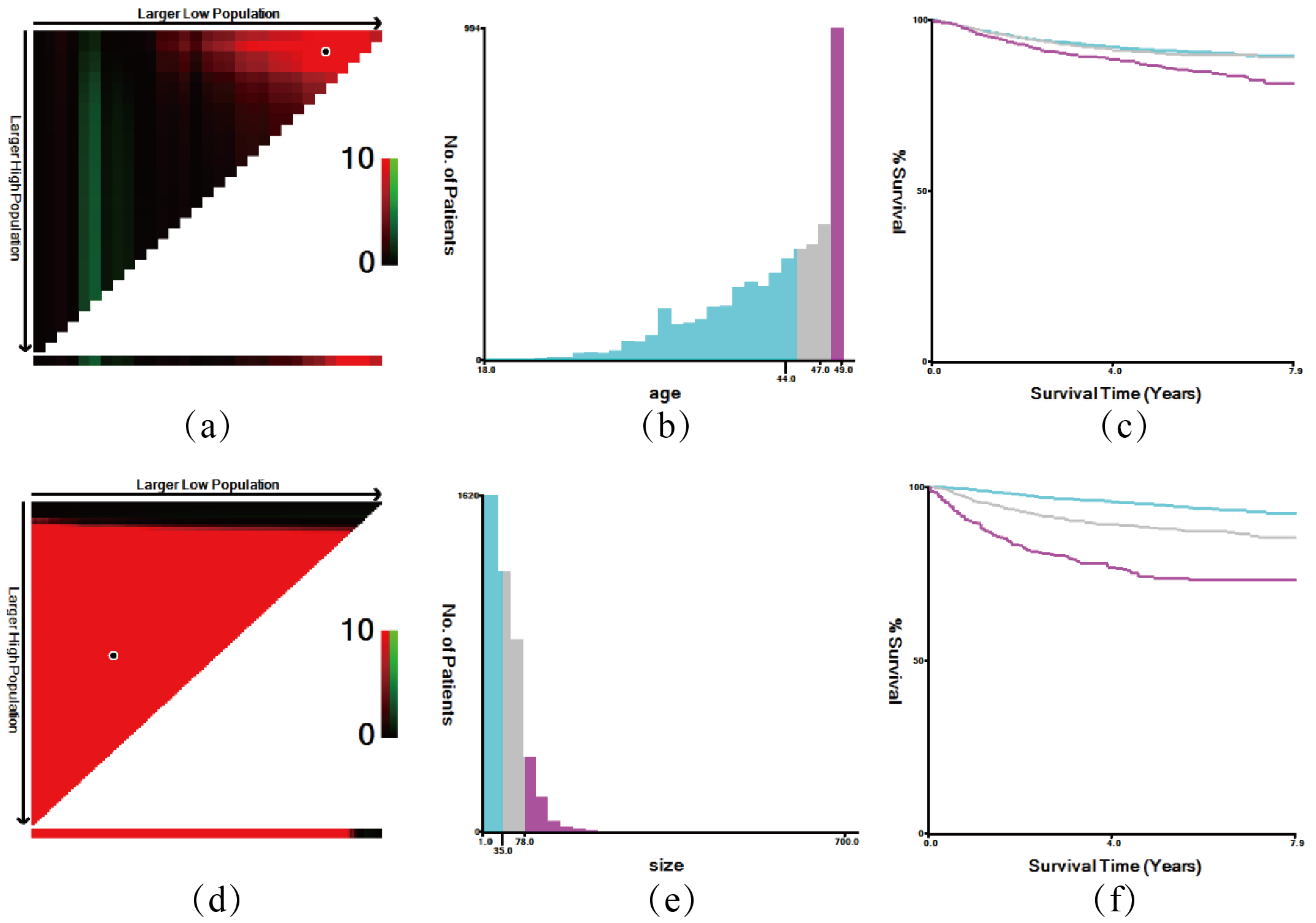


**Supplementary Fig. 2** The optimal thresholds for patients age (A-C) and tumor size (D-F) in the entire cohort were assessed by X-tile.

**Supplementary Table 1** Analysis of survival differences between non-surgical and surgical groups in EOEC cohort

| **Group** | **Cases (%)** | **1-year OS rate**  **(95% CI)** | **3-year OS rate**  **(95% CI)** | **5-year OS rate**  **(95% CI)** | ***P*-value^a^** |
| --- | --- | --- | --- | --- | --- |
| **Non surgical group** | 70 (1.6) | 79.8%  (70.4%-89.2%) | 71.6%  (60.6%-82.6%) | 64.2%  (51.5%-76.9%) | <0.001 |
| **Surgical group** | 4345 (98.4) | 99.8%  (99.6%-99.9%) | 92.6%  (91.8%-93.4%) | 90.2%  (89.2%-91.2%) |  |

**Note:** ^a^Log rank test of overall survival;

**Abbreviation:** CI, confidence interval.

**Supplementary Table 2** Clinicopathological characteristics of the external validation cohort

| **Variables** | **FAHCQMU cohort** | | **Variables** | **FAHCQMU cohort** | |
| --- | --- | --- | --- | --- | --- |
|  | **cases** | **%** |  | **cases** | **%** |
| **Age (years)** |  |  | **M stage** |  |  |
| <45 | 87 | 37.8 | M0 | 230 | 100.0 |
| 45-47 | 85 | 36.9 | M1 | 0 | 0.0 |
| 47-49 | 58 | 25.3 | **Tumor size (cm)** |  |  |
| **Race** |  |  | <3.6 | 172 | 74.8 |
| White | 0 | 0.0 | 3.6-7.8 | 58 | 25.2 |
| Black | 0 | 0.0 | >7.8 | 0 | 0.0 |
| Other | 230 | 100.0 | **SEER stage** |  |  |
| **Grade** |  |  | Localized | 197 | 85.7 |
| I | 81 | 35.2 | Regional | 33 | 14.3 |
| II | 119 | 51.7 | Distant | 0 | 0.0 |
| III | 30 | 13.1 | **Surgery** |  |  |
| IV | 0 | 0.0 | No | 0 | 0.0 |
| **T stage** |  |  | Yes | 230 | 100.0 |
| T1 | 190 | 82.6 | **Lymphadenectomy** |  |  |
| T2 | 39 | 17.0 | No | 5 | 2.2 |
| T3 | 1 | 0.4 | Yes | 225 | 97.8 |
| T4 | 0 | 0.0 | **Adjuvant treatment** |  |  |
| **N stage** |  |  | No/Unknown | 104 | 45.2 |
| N0 | 197 | 85.7 | Only radiotherapy | 70 | 30.4 |
| N1 | 18 | 7.8 | Only chemotherapy | 10 | 4.4 |
| N2 | 15 | 6.5 | Chemoradiotherapy | 46 | 20.0 |

**Abbreviations**: Grade I, well differentiated; Grade II, moderately differentiated; Grade III/IV, poorly differentiated or undifferentiated; Data are presented as number with percentage.

**Supplementary Table 3** Clinicopathological characteristics of the SEER cohort and the FAHCQMU cohort.

| **Variables** | **SEER cohort**  **[cases (%)]** | **FAHCQMU cohort**  **[cases (%)]** |
| --- | --- | --- |
| **Total** | 4345 | 230 |
| **Age (years)** |  |  |
| < 45 | 2290 (52.7) | 87 (37.8) |
| 45-47 | 1074 (24.7) | 85 (36.9) |
| 47-49 | 981 (22.6) | 58 (25.3) |
| **Race** |  |  |
| White | 3268 (75.2) | 0 (0.0) |
| Black | 324 (7.5) | 0 (0.0) |
| Other | 753 (17.3) | 230 (100.0) |
| **Grade** |  |  |
| I | 2428 (55.9) | 81 (35.2) |
| II | 1202 (27.7) | 119 (51.7) |
| III | 567 (13.0) | 30 (13.1) |
| IV | 148 (3.4) | 0 (0.0) |
| **T stage** |  |  |
| T1 | 3555 (81.8) | 190 (82.6) |
| T2 | 313 (7.2) | 39 (17.0) |
| T3 | 430 (9.9) | 1 (0.4) |
| T4 | 47 (1.1) | 0 (0.0) |
| **N stage** |  |  |
| N0 | 3968 (91.4) | 197 (85.7) |
| N1 | 232 (5.3) | 18 (7.8) |
| N2 | 145 (3.3) | 15 (6.5) |
| **M stage** |  |  |
| M0 | 4172 (96.0) | 230 (100.0) |
| M1 | 173 (4.0) | 0 (0.0) |
| **Tumor size (cm)** |  |  |
| < 3.6 | 1098 (25.3) | 172 (74.8) |
| 3.6-7.8 | 2979 (68.5) | 58 (25.2) |
| > 7.8 | 268 (6.2) | 0 (0.0) |
| **SEER stage** |  |  |
| Localized | 3196 (73.6) | 197 (85.7) |
| Regional | 954 (22.0) | 33 (14.3) |
| Distant | 195 (4.4) | 0 (0.0) |
| **Lymphadenectomy** |  |  |
| No | 1813 (41.7) | 5 (2.2) |
| Yes | 2532 (58.3) | 225 (97.8) |
| **Adjuvant treatment** |  |  |
| No/Unknown | 3070 (70.7) | 104 (45.2) |
| Only radiotherapy | 431 (9.9) | 70 (30.4) |
| Only chemotherapy | 389 (9.0) | 10 (4.4) |
| Chemoradiotherapy | 445 (10.4) | 46 (20.0) |

**Abbreviations**: Grade I, well differentiated; Grade II, moderately differentiated; Grade III/IV, poorly differentiated or undifferentiated.

**Supplementary Table 4** Analysis of survival differences between high-, intermediate-, and low-risk groups in the SEER cohort and the FAHCQMU cohort.

| **Cohort** | **Group** | **3-year OS rate**  **(95% CI)** | 1. **year OS rate**   **(95% CI)** | ***P*-value^a^** |
| --- | --- | --- | --- | --- |
| Training Cohort^b^  (n = 3044) | High-risk group  (n =306) | 60.7%  (55.0%-66.4%) | 53.8%  (47.7%-59.9%) | <0.001 |
|  | Intermediate-risk group  (n = 623) | 90.8%  (88.4%-93.2%) | 85.9%  (82.8%-89.0%) |  |
|  | Low-risk group  (n = 2115) | 97.7%  (97.1%-98.3%) | 96.4%  (95.4%-97.4%) |  |
| Internal Validation Cohort^b^  (n = 1301) | High-risk group  (n =124) | 58.1%  (49.3%-66.9%) | 51.2%  (41.6%-60.8%) | <0.001 |
|  | Intermediate-risk group  (n = 232) | 89.1%  (84.8%-93.4%) | 86.2%  (81.3%-91.1%) |  |
|  | Low-risk group  (n = 945) | 98.1%  (97.1%-99.1%) | 97.0%  (95.8%-98.2%) |  |
| External Validation Cohort^c^  (n = 230) | High-risk group  (n = 6) | 83.3%  (53.5%-100.0%) | 55.6%  (26.2%-85.0%) | <0.001 |
|  | Intermediate-risk group  (n = 35) | 94.3%  (86.7%-99.9%) | 94.3%  (86.7%-99.9%) |  |
|  | Low-risk group  (n = 189) | 98.9%  (97.5%-99.9%) | 98.9%  (97.5%-99.9%) |  |

**Note:** ^a^Log rank test of OS; ^b^SEER cohort; ^c^FAHCQMU cohort.

**Abbreviation:** CI, confidence interval.


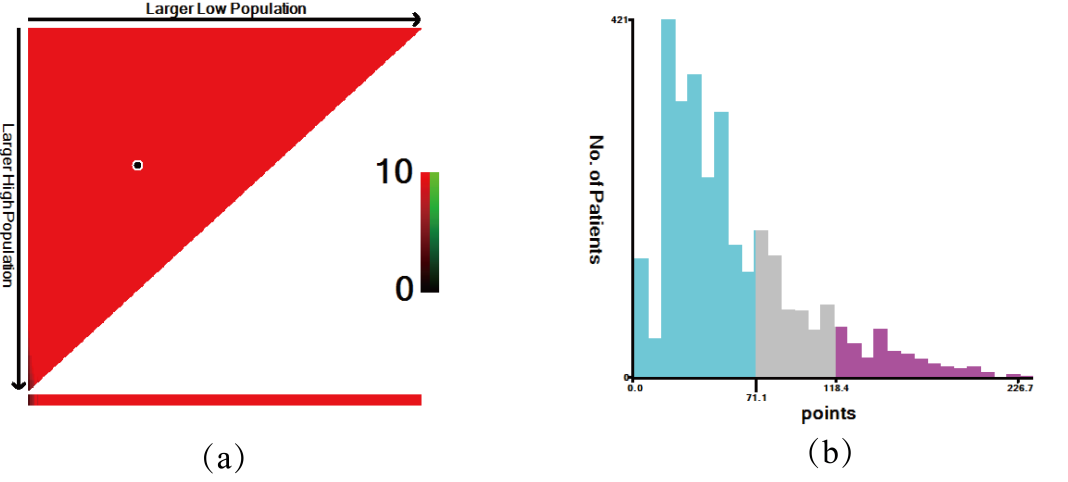


**Supplementary Fig. 3** Cut-off values calculated by X-tile software (A) and (B). The determined cut-off value was 71.1 and 118.4, categorizing EOEC patients into high-risk group (total score > 118.4 pts), intermediate-risk group (total score: 71.1-118.4 pts), and low-risk group (total score < 71.1 pts).
