# Supplementary material for: Comparing survival outcomes between surgical and non-surgical treatments in patients with early-onset endometrial cancer and developing a nomogram to predict survival: a study based on Eastern and Western data sets
Source: World J Surg Oncol. 2025 May 11;23:184. doi: 10.1186/s12957-025-03825-y (PMC12067707; doi:10.1186/s12957-025-03825-y)
Supplement: Supplementary file 3 — Supplementary Material 3. [file 12957_2025_3825_MOESM3_ESM.docx]

**Supplementary materials**

Description: Related Computerized Programs for Nomogram with R

**APPENDIX**

**Related computerized programs for nomogram with R**

**for OS nomogram**

library(survival)

library(rms)

dd<-datadist(data)

options(datadist="dd")

f<-cph(Surv(time,status) ~ Age+Size+Race+Grade+Tstage+Surgery, data=data, x=TRUE, y=TRUE, surv=TRUE)

survival<-Survival(f)

Survival1<-function(x)survival(36,x)

Survival2<-function(x)survival(60,x)

nom<-nomogram(f,fun=list(survival1,survival2),fun.at

= c(0.05,seq(0.1,0.9,by=0.1),0.95),funlabel = c('3-year survival','5-year survival'))

plot(nom)

**For CSS nomogram**

library(survival)

library(rms)

dd<-datadist(data)

options(datadist="dd")

f<-cph(Surv(time,CSS) ~ Age+Size+Race+Grade+Tstage+Surgery, data=data, x=TRUE, y=TRUE, surv=TRUE)

survival<-Survival(f)

Survival1<-function(x)survival(36,x)

Survival2<-function(x)survival(60,x)

nom<-nomogram(f,fun=list(survival1,survival2),fun.at

= c(0.05,seq(0.1,0.9,by=0.1),0.95),funlabel = c('3-year survival','5-year survival'))

plot(nom)

**For computing the C-index and 95% *CI* in training cohort and validation**

**cohort**

library(survival)

library(rms)

fit<-coxph(Surv(time,status)~Age+Size+Race+Grade+Tstage+Surgery,data = Ytraindata)

survConcordance(Surv(Ytraindata$time,Ytraindata$status)~predict(fit,Ytraindata))

survConcordance(Surv(Ytestdata$time,Ytestdata$status)~predict(fit,Ytestdata))

**For ROC curve for OS and CSS in training cohort**

library(survivalROC)

colnames(seer) <- c("id","survival_time","status","risk_score","predict_time")

predict_time<-12*3

myroc<-survivalROC(Stime=seer$survival_time,status=seer$status, marker=seer$risk_score, predict.time=predict_time,method="KM")

pdf("ROC_3-year_OS_in_Training_set.pdf")

plot(myroc$FP,myroc$TP,type="l",xlim=c(0,1),ylim=c(0,1),col="blue",

xlab="FP",ylab="TP",lwd=2.5,main=paste("3-year Survival","AUC=",round(myroc$AUC,3)))

abline(0,1)

dev.off()

colnames(seer) <- c("id","survival_time","CSS","risk_score","predict_time")

predict_time<-12*3

myroc<-survivalROC(Stime=seer$survival_time,status=seer$CSS, marker=seer$risk_score, predict.time=predict_time,method="KM")

pdf("ROC_3-year_CSS_in_Training_set.pdf")

plot(myroc$FP,myroc$TP,type="l",xlim=c(0,1),ylim=c(0,1),col="red",

xlab="FP",ylab="TP",lwd=2.5, main=paste("3-year Survival","AUC=",round(myroc$AUC,3)))

abline(0,1)

dev.off()

**For ROC curve for OS and CSS in validation cohort**

library(survivalROC)

colnames(seer) <- c("id","survival_time","status","risk_score","predict_time")

predict_time<-12*3

myroc<-survivalROC(Stime=seer$survival_time,status=seer$status, marker=seer$risk_score, predict.time=predict_time,method="KM")

pdf("ROC_3-year_OS_in_Validation_set.pdf")

plot(myroc$FP,myroc$TP,type="l",xlim=c(0,1),ylim=c(0,1),col="blue",

xlab="FP",ylab="TP",lwd=2.5,main=paste("3-year Survival","AUC=",round(myroc$AUC,3)))

abline(0,1)

dev.off()

colnames(seer) <- c("id","survival_time","CSS","risk_score","predict_time")

predict_time<-12*3

myroc<-survivalROC(Stime=seer$survival_time,status=seer$CSS, marker=seer$risk_score, predict.time=predict_time,method="KM")

pdf("ROC_3-year_CSS_in_Validation_set.pdf")

plot(myroc$FP,myroc$TP,type="l",xlim=c(0,1),ylim=c(0,1),col="red",

xlab="FP",ylab="TP",lwd=2.5, main=paste("3-year Survival","AUC=",round(myroc$AUC,3)))

abline(0,1)

dev.off()

**For calibration curve for training cohort**

library(survival)

library(rms)

f1<-cph(Surv(Ytraindata$time,Ytraindata$status==1)~Age+Size+Race+Grade+Tstage+Surgery,data = Ytraindata,x=TRUE,y=TRUE,surv=TRUE,time.inc=3*12)

cal<-calibrate(f1, cmethod = 'KM', method = "boot", u=3*12, m=180, B=1000)

plot(cal, lwd=1, lty=1, errbar.col=c(rgb(0,0,0,maxColorValue = 255)), xlim = c(0.8,1), ylim = c(0.7,1), xlab ="Nomogram Predicted Survival", ylab="Actual

Survival", col=c(rgb(255,0,0, maxColorValue =255)))

abline(0,1,lty = 3, lwd = 2, col = c(rgb(0,118,192,maxColorValue=255)))

lines(cal[,c('mean.predicted','KM')], type = 'b',lwd = 2, col = c(rgb(192,98,83,maxColorValue = 255)), pch = 16)

**For predictions of the validation cohort**

library(survival)

library(rms)

f<-cph(Surv(time,status)~Age+Size+Race+Grade+Tstage+Surgery,data = Ytraindata)

f<-predict(f, newdata = Ytestdata)

predictions<-predict(f, newdata = Ytestdata)

predictions

**For calibration curve for validation cohort**

f2<-cph(Surv(Ytestdata$time,Ytestdata$status,type = "right")~predictions, x=T,y=T,surv=T, time.inc =3*12)

validate(f2,method = "boot",B=1000,dxy=T,u=3*12)

cal<-calibrate(f2,cmethod = 'KM',method="boot",u=3*12,m=100,B=1000)

plot(cal,lwd=1,lty=1,errbar.col=c(rgb(0,0,0,maxColorValue = 255)),xlim = c(0.8,1),ylim = c(0.7,1),xlab ="Nomogram Predicted Survival ",ylab="Actual Survival",col=c(rgb(255,0,0,maxColorValue =255)))

abline(0,1,lty = 3,lwd = 2,col = c(rgb(0,118,192,maxColorValue=255)))

lines(cal[,c('mean.predicted','KM')], type = 'b',lwd = 2, col = c(rgb(192,98,83, maxColorValue = 255)),pch = 16)

**For computing the C-index and 95% *CI* of different models for OS**

AJCC TNM stage

library(survival)

library(rms)

fit1<-coxph(Surv(time,status)~Tstage+Nstage+Mstage,data = Ytraindata)

survConcordance(Surv(Ytraindata$time,Ytraindata$status)~predict(fit1,Ytraindata))

survConcordance(Surv(Ytestdata$time,Ytestdata$status)~predict(fit1,Ytestdata))

SEER stage

library(survival)

library(rms)

fit1<-coxph(Surv(time,status)~SEERstage,data = Ytraindata)

survConcordance(Surv(Ytraindata$time,Ytraindata$status)~predict(fit1,Ytraindata))

survConcordance(Surv(Ytestdata$time,Ytestdata$status)~predict(fit1,Ytestdata))

**For computing the C-index and 95% *CI* of different models for CSS**

AJCC TNM stage

library(survival)

library(rms)

fit1<-coxph(Surv(time,CSS)~Tstage+Nstage+Mstage,data = Ytraindata)

survConcordance(Surv(Ytraindata$time,Ytraindata$CSS)~predict(fit1,Ytraindata))

survConcordance(Surv(Ytestdata$time,Ytestdata$CSS)~predict(fit1,Ytestdata))

SEER stage

library(survival)

library(rms)

fit1<-coxph(Surv(time,CSS)~SEERstage,data = Ytraindata)

survConcordance(Surv(Ytraindata$time,Ytraindata$CSS)~predict(fit1,Ytraindata))

survConcordance(Surv(Ytestdata$time,Ytestdata$CSS)~predict(fit1,Ytestdata))
